# Knowledge, attitudes, and practices on camel respiratory diseases and conditions in Garissa and Isiolo, Kenya

**DOI:** 10.3389/fvets.2022.1022146

**Published:** 2022-11-29

**Authors:** Joseph Othieno, Obadiah Njagi, Sophie Masika, Michael Apamaku, Evans Tenge, Bridgit Mwasa, Peter Kimondo, Emma Gardner, Sophie Von Dobschuetz, Joseph Muriira, Ben Adul, Lawrence Mwongela, Haret A. Hambe, Thomas Nyariki, Folorunso O. Fasina

**Affiliations:** ^1^Directorate of Veterinary Services, Ministry of Agriculture, Livestock, Fisheries and Cooperatives, Nairobi, Kenya; ^2^Emergency Center for Transboundary Animal Diseases, Food and Agriculture Organization of the United Nations, Nairobi, Kenya; ^3^Food and Agriculture Organization of the United Nations, Rome, Italy; ^4^Ministry of Agriculture, Livestock and Fisheries, Isiolo County, Kenya; ^5^Ministry of Agriculture, Livestock and Fisheries, Garissa County, Kenya; ^6^Department of Veterinary Tropical Diseases, University of Pretoria, Pretoria, South Africa

**Keywords:** camel respiratory diseases, knowledge, attitudes, practices, Kenya, risk communication and community engagement

## Abstract

**Background:**

Livestock farmers' attitudes, practices, and behaviors are major factors in infection prevention and control of animal diseases. Kenya has the fourth largest global camel population, and the industry has grown over the last two decades, transforming beyond the traditional camel-keeping areas to include peri-urban camel trade and value chain growth. The dromedary camel is resilient, and it is a preferred species in the arid and semi-arid areas (ASALs) of Kenya. However, it still faces many health and production challenges; to identify infection drivers and risky behaviors for camel respiratory illnesses and conditions in Kenya, we conducted a knowledge, attitudes, and practices (KAP) survey.

**Method:**

Using a set of tools (questionnaires, key informant interviews, and focus group discussions), we interviewed camel owners, herders, agro-veterinary outlets, and other relevant value chain stakeholders in Garissa and Isiolo counties (*n* = 85). Data were analyzed using descriptive and analytic statistics.

**Results:**

Most camel owners/herders are male and most are relatively uneducated (85.5%). The camels were used primarily for milk and meat production, income generation, and transport. Larger herd sizes (>30 camels) and owner/herder's lack of formal education are risk factors for owner-reported respiratory illnesses in camels. Major clinical signs of respiratory conditions were coughing (85.7%), nasal discharge (59.7%), and fever (23.4%). Diseases, lack of feeds, theft, and marketing challenges are the major constraints to camel production in Kenya. Owners-herders use drugs indiscriminately and this may contribute to antimicrobial resistance challenges.

**Conclusion:**

Practitioners in the camel value chain want more commitment from the government and animal health officials on support services and access to veterinary services. Watering points, grazing areas, and marketing points are the primary areas for congregating camels and have a significant potential for disease spread. Kenya camels have a massive capacity for rural and ASALs' livelihoods transformation but the identified health challenges, and other issues must be addressed. Further studies on the Kenyan camels' respiratory microbial ecology are important to understand microbial risks and reduce the burden of zoonotic infections. Intensification of risk communication and community engagement, and messaging targeted at behavior change interventions should be directed at camel value chain actors.

## Introduction

The dromedary camel (*Camelus dromedarius*) is an important species in the Arid and Semi-Arid Lands (ASALs) agro-ecosystems of the world (1). Over 80% of the world's camel population lives in Africa with 60% of these in the Horn of Africa where they make a significant part of export, cross-border, and in-country trade, as well as food security and livelihoods of local communities (1–3). Additionally, the species is socio-culturally significant to some communities in matters, such as conflict resolution and dowry payment (4).

Kenya has the fourth largest camel population in the world, with an in-country estimate of 4.6 million in 2019 (5, 6). The camel industry in Kenya has grown steadily over the last two decades with the growth of peri-urban trade and expansion of camel keeping beyond the traditional areas (7, 8). The camel is becoming a preferred species for resilient livelihoods among pastoralist communities due to its superior adaptability to frequent droughts in the face of increasing climate variability (9).

This study was a follow-on from investigations into mass deaths of camels in northern Kenya and the greater Horn of Africa in early 2020. A respiratory syndrome characterized by nasal discharge, coughing, difficulty in breathing, and death affecting young camels had been reported in Marsabit, Wajir, Isiolo, and Garissa counties (10). The event raised speculations that the Middle East Respiratory Syndrome Corona virus (MERS-CoV), a zoonotic betacoronavirus, might have been the cause of the outbreak (11). Epidemiological and laboratory investigations, however, confirmed that it is a bacterial disease caused by Mannheimia haemolytica (10–12).

The emergence of human cases of MERS-CoV in Saudi Arabia in 2012, with subsequent evidence pointing to dromedary camels as a reservoir host for the virus, posed a threat to camel exports from the Horn of Africa to the Middle East (13–15). Growing evidence from phylogenetic studies on MERS-CoV isolates from the continent, however, shows that the lineages of the virus circulating in Africa are distinctly different from those circulating in humans and camels in the Middle East (16–19). This suggested that camel imports from Africa were not significant for the circulation of the virus in camels and humans in the Middle East (16).

The zoonotic potential of MERS-CoV clades circulating in Africa, however, remains a concern based on serological evidence of spillover of virus to humans at the camel–human interface and on infectivity studies, in tissue culture, of virus isolates from the region (19–22). The emergence of COVID-19 pandemic in 2019, caused by another betacoronavirus, the Severe Acute Respiratory Syndrome Coronavirus (SARS-CoV-2), brought to the fore the need for accurate public information, education, and communication about camel respiratory conditions in relation to camel productivity and public health concerns. It is within this context that the Directorate of Veterinary Services, the County governments of Garissa and Isiolo, and the Food and Agriculture Organization of the United Nations (FAO) collaborated to undertake a Knowledge, Attitudes, and Practices (KAP) study on camel respiratory conditions among camel value-chain actors in the two counties. The purpose of the study was to provide data and evidence for the development of Information, Education and Communication materials (IECs) as part of communication interventions on camel respiratory conditions.

## Materials and methods

**Figure 1:**
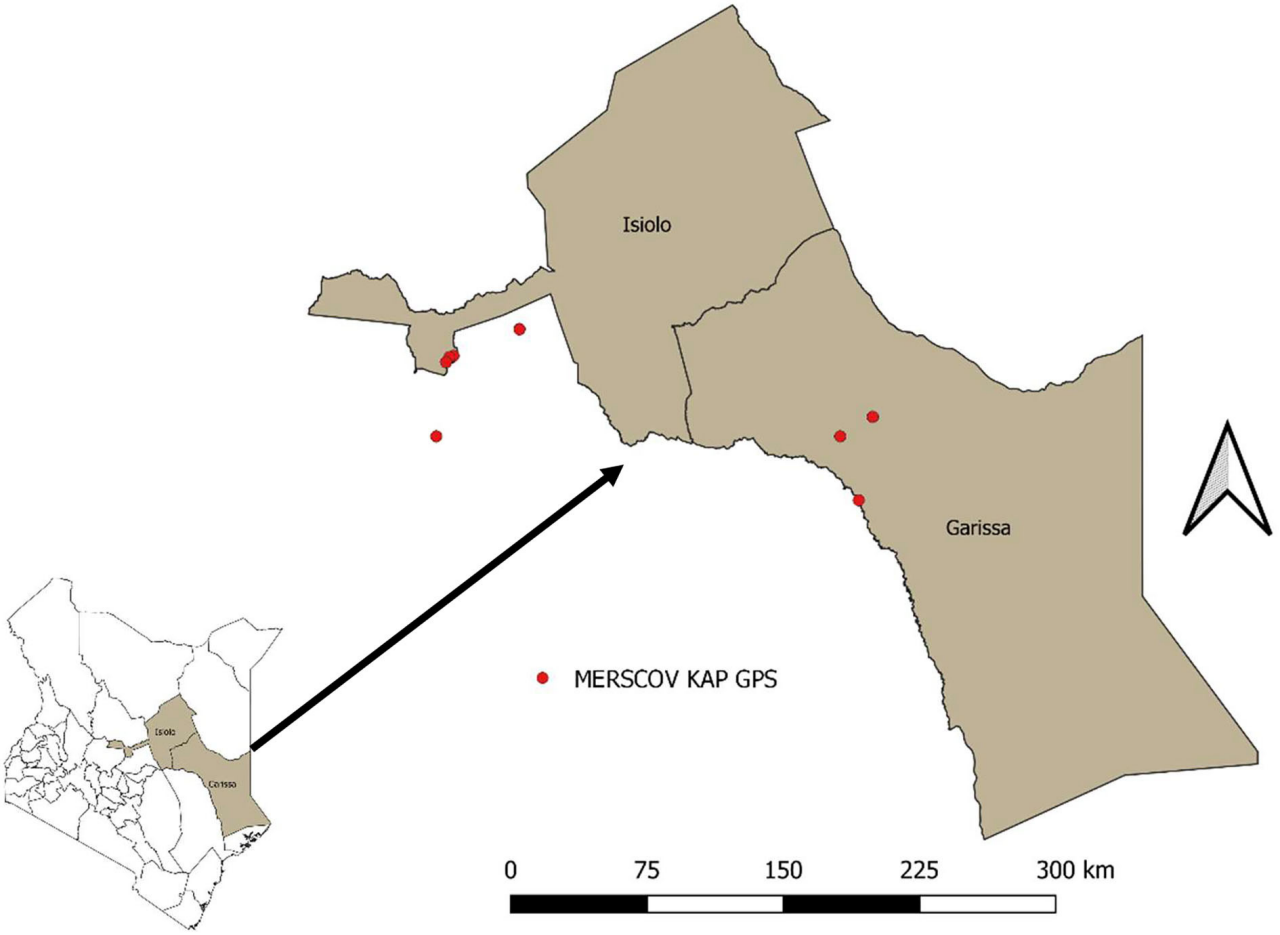
Map of areas where the survey was done.

A cross-sectional KAP survey was carried out in two counties of Isiolo and Garissa, Kenya (Figure 1) in the month of October 2020. The study involved camel owners, camel herders, community opinion leaders, and animal health professionals, as well as agro-veterinary shop owners. Several tools were employed in the study: questionnaires, key informant interviews, focus group discussions, and checklists for observations.

### Questionnaire development and administration

A questionnaire was developed through stakeholders' consultations and desk review. Specifically, responsible staff of the Directorate of Veterinary Services, Ministry of Agriculture, Livestock, Fisheries and Cooperatives (DVS MoALFC), and Food and Agriculture Organization of the United Nations (FAO) facilitated stakeholders meeting through which a list of issues that may play roles or influence respiratory disease incidences in camel were generated, through an iterative process, repetitive questions, and redundant issues were removed. The final list of questions was harmonized to produce a list of questions in the questionnaire (Supplementary material 1). This was pre-tested among five camel herders who did not form part of the interviewed participants. Based on the feedback, the questionnaire was adjusted, and the final version was used to conduct an interview in the field through administration to camel owners and camel herders. The questionnaire had three categories of respondents including camel owners, camel herders, and camel owners herding their own camels. The questionnaire was used to gather general information on camel health issues and specifically respiratory diseases. It was structured into four sections: demographics, knowledge, attitudes, and practices. The outputs were knowledge levels on the benefits of camels, constraints to camel keeping, general diseases and their causes, and clinical presentations of respiratory conditions (Supplementary material 1). The attitudes toward camel health issues were documented, and various practices among camel owners and herders were listed.

The second questionnaire developed was the Key Informant Interview (KII), which was directed to the animal health service providers including the County Veterinary Officers, Animal Health Assistants (AHAs), animal production officers, and agro-veterinary shop owners (Supplementary material 2). Camel traders were also interviewed based on their knowledge of camel health issues learned over time. In addition, Focus Group Discussions (FGDs) were held with four Camel Association groups using a semi-structured key informant guide (Supplementary material 3). The questions in the Supplementary material 3 were aimed at triangulating the responses from the individual farmers and generating opinions on the relationship with the government, camel farming, welfare, and the challenges impacting camel farming in Kenya. Using these semi-structured tools, qualitative and quantitative data were collected. While the questionnaire survey provided a quantitative or numeric description of trends, attitudes, or opinions of the value chain stakeholders across the selected population, it also triggered some issues that needed some in-depth analyses. The key informant interviews provided the follow-up in-depth discussions with persons who were considered to have expert knowledge, in order to validate the earlier opinions. The focus group discussions were held to provide an open-ended cross-validations of the survey and to check whether the individual value chain perspective was similar or variant with the group views.

A total of 85 questionnaires were administered to camel owners and herders in Isiolo (*n* = 44) and Garissa (*n* = 41). Both counties were selected purposively from the list of counties with high camel populations (23). Villages were selected randomly from the list of villages per county. In Isiolo, the questionnaires were administered in Idafin, LMD, Bullo, Endomuru, Akadeli, Haidaffi, and Burrat villages. Similarly, in Garissa, the questionnaires were administered in the villages of Abdisamid, Shimbiry, and Bula-Rahma. In addition, 28 key informant interviews were carried out in the two counties. The participants were 16 veterinarians/animal health assistants (AHAs), 10 camel traders, one member of the Camel Association in Isiolo, and an official from the Livestock Market Trust (two opinion leaders). Four focused groups' discussions were held, two in Endomoru in Isiolo and another two in Bulla-Gawan, Garissa.

### Data analysis

Data were entered into and filtered in Microsoft Excel v2016 (Microsoft Corporation, Redmond, Washington, USA). The data in the spreadsheet were transmitted into the IBM^®^ SPSS^®^ Statistics version 20 for analysis. Descriptive statistics including frequencies and exact confidence intervals at a 95% level were calculated. The leading constraint to camel production was determined using serial positioning. To determine the association between different variables chi-square tests were performed with a *p*-value set at 0.05. A pairwise correlation was determined among relevant variables with a significant association set at 0.05. Risk-based (sub-population level and population level risks in percentages) and odds-based (conditional maximum likelihood estimate of Odds Ratio) estimates of variables were carried out using the two-by-two table in OpenEpi^®^ (24).

## Results

### Demographics

Out of the 85 individual respondents, 71 (83.5%) were males and 14 (16%) were females. Most of the respondents identified as Muslim (94.1%) while the remaining 5.9% identified as Christian. A total of 70.6% of the respondents were in the age category of 26–55 years. The age distribution of the other respondents is indicated in Table 1. The majority of the respondents had not received a formal education, with 77.6% not having attended school, and an additional of 8.2% having not completed primary school. In terms of herd size, 61.2% of those interviewed had more than 30 camels (classified as large herd), 30.6% have medium herd sizes (6–30 camels), and only 8.2% have small herds (1–5 camels). Furthermore, the majority of the respondents were camel owners (61.2 %), and 17.6% were purely camel herders, while the remainder 21.2% herded their own camels (Table 1).

**Table 1:** Demographic variables of the respondents.

**Demographic variable**	**Number**	**Percentage (%)**
**Gender**	**85**	**100**
Male	71	83.5
Female	14	16.5
**Age of respondents**	**85**	**100**
18–25	7	8.2
26–35	21	24.7
36–45	25	29.4
46–55	14	16.5
56–65	8	9.4
66>	10	11.8
**Education level of respondent**	**85**	**100**
None/never been to school	66	77.6
Primary incomplete	7	8.2
Primary complete	8	9.4
Secondary incomplete	2	2.4
Secondary complete	1	1.2
Tertiary incomplete	1	1.2
**Religion of respondent**	**85**	**100**
Christian	5	5.9
Muslim	80	94.1
**Herd size**	**85**	**100**
Small (1–5 camels)	7	8.2
Medium (6–30 camels)	26	30.6
Large (>30 camels)	52	61.2
**Respondent type**	**85**	**100**
Owner	52	61.2
Herder	15	17.6
Both owner and herder	18	21.2

Bold values mean cumulative here.

Using the risk and odds-based estimates, the population-level risk for respiratory conditions in the studied Kenya's camel is 91.7%. The risk of respiratory conditions in the male camel (91.6%) is slightly less than in the female (92.3%) although the odds of the risk is 0.90 in male vs. female (*p* = 1.00). Similar profiles exist for differences between Isiolo (90.9%) and Garissa (92.5%) counties (OR = 0.81; *p* = 0.81). The medium-sized herd is 3-fold less likely and has a 9.5% less risk of contracting respiratory conditions (*p* = 0.21) (Table 2). Camel herds of individuals with no formal or incomplete primary education are 3-fold more likely and have 9.8% more risk of respiratory conditions compared to those who have completed primary schooling or more (*p* = 0.32). Compared to the herds managed by herders, herds of owners and those of individuals who combined the role of owner-herder are ~0.66-fold (*p* = 0.78) and 0.55-fold (*p* = 0.65) less likely to have respiratory conditions, respectively (Table 2). Based on the feedback from the respondents, the outcomes of respiratory conditions or diseases in the camel herds may lead to recovery (14.3%), death (41.5%), or uncertain situation of death or recovery (44.2%).

**Table 2:** Risk and odds-based estimates of respiratory illnesses and conditions in Kenya camels.

**Variable**		**Respiratory condition present**	**Respiratory condition absent**	**Sub-population level risk (%)**	Population-level risk^*^(%)	CMLE Odds ratio^**^	***P*-value**
Gender	Male	65	6	91.6	91.7	0.90 (0.03; 6.89)	1.00
	Female	12	1	92.3		1.00	NA
County	Isiolo	40	4	90.9	91.7	0.81 (0.14; 4.18)	0.81
	Garissa	37	3	92.5		1.00	NA
Herd size	Small	7	0	NA	90.9	–	–
	Medium	22	4	84.6		0.35 (0.06; 1.82	0.21
	Large	48	3	94.1		1.00	NA
Education	No formal education/incomplete primary	67	5	93.1	91.7	2.64 (0.32; 15.46)	0.32
	Complete primary up to tertiary	10	2	83.3		1.00	NA
Responsibility to camel	Owner	46	5	90.2	90.9	0.66 (0.02; 5.29)	0.78
	Herder	14	1	93.3		1.00	NA
	Owner–herder	17	1	94.4	91.3	0.55 (0.02; 4.30)	0.65

* Risk-based estimates.

** Odds-based estimates.

CMLE, Conditional maximum likelihood estimate of Odds Ratio.

Based on the feedback from the respondents, the outcomes of respiratory conditions or diseases in the camel herds may lead to recovery (14.3%), death (41.5%), or uncertain situation of death or recovery (44.2%). NA, Not applicable.

Using serial positioning, camel diseases were ranked as the greatest constraint to camel production, followed by feeds, marketing, and then theft (Table 3). Other issues that flagged up as constraints were predation, water scarcity, injuries and accidents to animals, poor farming and management system, cost of maintaining the herders, hardship experienced with herding, drought, and land disputes in that order.

**Table 3:** Constraints to camel production.

**Constraints**	**Position 1 (%)**	**Position 2 (%)**	**Position 3 (%)**	**Position 4 (%)**
Diseases	**53.6**	21.9	16.3	14.3
Feeds	14.3	**37.9**	16.3	0
Theft	17.9	21.9	12.2	**14.3**
Marketing	4.8	9.6	**30.6**	21.4

Bold values mean priority selection here.

### Knowledge and awareness

**Figure 2:**
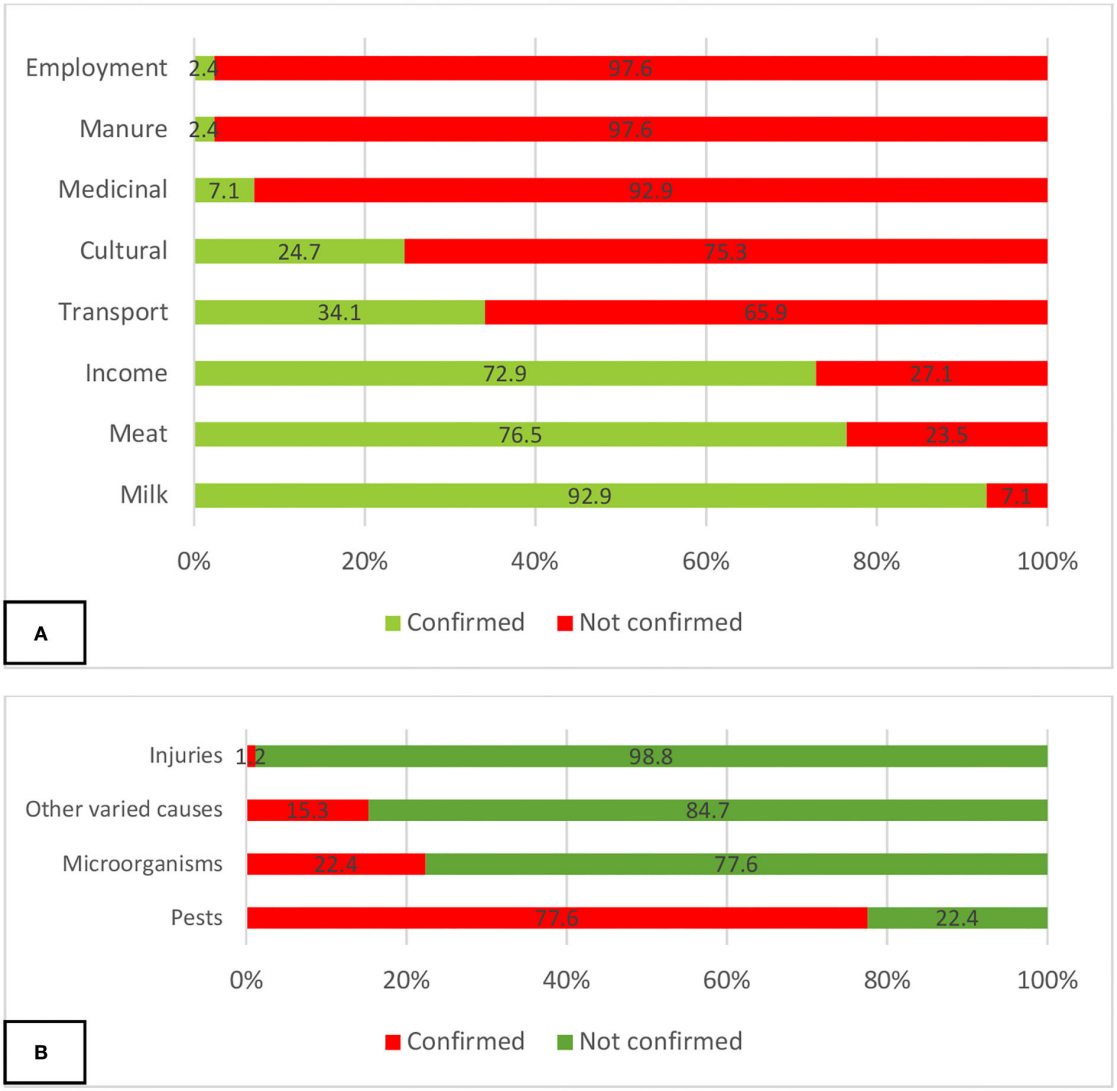
Self-reported **(A)** benefits of camel rearing and **(B)** leading causes of camel diseases.

The respondents confirmed that camel farming and management are beneficial and the lead reason why they farmed camel include the following: provision of milk (92.9%), meat (76.5%), income (72.9%), transport (34.1%), and for cultural activities for example during dowry payment (24.7%) (Figure 2A).

In terms of causes of diseases in camels, although the owners and herders were not able to mention specific diseases, they were aware of the causes of diseases in camels based on interactions with their animal health officials. Pests (mosquitoes, tsetse flies, and ticks) were reported by 77.6% of the respondents, microorganisms by 22.4%, injuries by 1.2%, and other causes mentioned by 15.3% (Figure 2B).

The majority of the respondents (90.6 %) reported that their herds had suffered from respiratory conditions, especially during the rains. The following clinical signs have been observed as predictors of respiratory conditions and diseases in camels: coughing (85.7%), nasal discharge (59.7%), fever (23.4%), loss of appetite (20.8%), and enlarged lymph nodes (19.5%) as the most common signs as listed by camel keepers in the two counties (Table 4). Other signs and symptoms included body weakness (15.6%), weight loss (14.3%), recumbency (11.7%), drop in milk production (9.1%), excessive lacrimation (tears) (9.1%), sneezing (7.8%), enlarged abdomen (6.5%), shivering (6.5%), difficulty in breathing (5.2%), sudden death (2.6%), abortion (2.6%), and foaming in the mouth (1.3%) (Table 4). Difficulty in breathing was moderately positively correlated with foaming in the mouth (*p* < 0.05). Weak positive correlations were observed between a drop in milk production and abortion; recumbency and foaming in the mouth; inappetence and sudden death; enlarged lymph nodes and excessive lacrimation; excessive lacrimation and foaming in the mouth; weight loss and drop in milk production; weight loss and fever; shivering and sudden death; as well as sneezing and fever (*p* < 0.05). However, a moderate negative correlation was observed between nasal discharge and weight loss, but a weak negative correlation was observed between nasal discharge and sneezing and between coughing and an enlarged abdomen (*p* < 0.05) (Table 4).

**Table 4:** Symptoms and clinical signs as predictors of respiratory diseases in camels.

	**A**	**B**	**C**	**D**	**E**	**F**	**G**	**H**	**I**	**J**	**K**	**L**	**M**	**N**	**O**	**P**	**Q**
A	1.000																
B	−0.125	1.000															
C	0.013	−0.008	1.000														
D	0.123	0.185	−0.179	1.000													
E	0.067	0.088	0.021	0.030	1.000												
F	−0.039	−0.194	0.116	0.124	−0.039	1.000											
G	−0.044	0.010	0.208	−0.185	0.095	−0.061	1.000										
H	0.092	0.043	0.223	−0.058	0.490^*^	−0.019	0.315^*^	1.000									
I	0.161	−0.023	0.169	0.298^*^	0.128	−0.053	0.023	0.362^*^	1.000								
J	–0.445^*^	0.037	0.244	−0.113	−0.098	0.165	−0.153	−0.048	−0.133	1.000							
K	0.102	0.099	0.122	−0.000	−0.063	−0.044	0.066	−0.031	−0.086	0.040	1.000						
L	0.013	0.049	0.128	−0.127	−0.104	−0.072	0.175	−0.051	−0.015	−0.078	−0.117	1.000					
M	0.161	–0.305^*^	−0.167	0.069	−0.076	−0.053	0.023	−0.037	−0.103	−0.133	0.098	−0.015	1.000				
N	0.013	0.061	0.318^*^	−0.083	−0.039	−0.027	0.194	−0.019	−0.053	−0.069	0.288^*^	−0.072	−0.053	1.000			
O	−0.216	−0.023	0.169	−0.160	−0.076	0.231^*^	0.164	−0.037	−0.103	0.385^*^	−0.086	−0.015	−0.103	−0.053	1.000		
P	–0.371^*^	0.109	−0.154	−0.147	−0.070	−0.049	−0.109	−0.034	−0.095	−0.122	−0.079	−0.129	−0.095	−0.049	−0.095	1.000	
Q	−0.131	−0.081	0.012	−0.125	−0.133	−0.093	−0.111	−0.065	−0.073	0.297^*^	−0.025	−0.075	0.142	−0.093	0.142	0.295^*^	1.000

A, Nasal discharge; B, Coughing; C, Inappetence; D, Enlarged lymph nodes; E, Difficulty in breathing; F, Abortion; G, Recumbency; H, Foaming in the mouth; I, Excessive lacrimation (tears); J, Weight loss; K, Shivering; L, Body weakness; M, Enlarged abdomen; N, Sudden death; O, Drop in milk production; P, Sneezing; Q, Fever.

* *p*-value ≤ 0.05. Bold values mean significant here.

### Attitudes

Camels were considered to be hardy animals by 58.8% of the respondents, while 41.2% thought not. Respiratory diseases were thought to be common in young camels by 60% of respondents, 30.6% thought it was common in all ages while 23.5% thought it was common in older camels (Table 5). On seasonality of the occurrence of respiratory diseases, most respondents (56.5%) reported that they occur more during the rainy season. While others thought that it occurs in dry and cold seasons; 35.35 and 29.4%, respectively. Others thought it occurs throughout the year (11.8%) while 3.5% thought respiratory diseases were common during cultural occasions (Table 5). The community disease reporters and the animal health assistants were more available than the veterinarians to handle camel health. According to respondents, herbalists and elders, community disease reporters, and animal health assistants were the preferred health service providers for their camels (Table 5). In addition, various sources of information and indigenous knowledge on camel health exist, and interpersonal information sharing and radio remain the leading sources of information. The interpersonal channel typically occurs at the watering points, by elders, at the markets, and places of worships (Table 5). Camel owners and herders perceived that government support for camel production is wanting (64.7%) although 29.4% believed that there is some government support.

**Table 5:** Attitudes and disposition to camel diseases and information sources.

**Variable**	**Classification**	**Frequency**	**Percentage**
Hardiness of camel	Yes	50	58.8
	No	35	41.2
Age predisposition for respiratory diseases (*n* = 85)	Young camels	51	60.0
	All ages	26	30.6
	Old camels	20	23.5
	Lactating	8	9.4
	Pregnant	6	7.1
Seasonal predisposition to respiratory diseases (*n* = 85)	Rainy season	48	56.6
	Cold season	30	35.3
	Dry season	25	29.4
	All the year round	10	11.8
	More during cultural occasion	3	3.5
Availability of animal health officials (*n* = 84)	Community disease reporter^*^	22	26.2
	Animal health assistant	21	25.0
	Veterinarian	14	16.7
Preferred service provider (*n* = 83)	Herbalist/elders	24	28.9
	Community disease reporter	20	24.1
	Animal health assistant	14	16.9
Level of government support (*n* = 85)	No support	55	64.7
	Little support	25	29.4
	Sufficient support	3	3.5
	I don't know	1	1.2
Preferred source of information (*n* = 83)	Interpersonal communication with fellow herd owner	24	28.9
	Radio	16	19.3
	Herbalist	11	13.3
	Agro-veterinary shop owner	7	8.4
	Community opinion leader	6	7.2
	Chief “*barazas*”^**^	5	6.0
	Veterinarian/animal health assistant	4	4.8
	Mobile phones	3	3.6
	Self-motivated learning	2	2.4
	Community disease reporter	2	2.4
	Training	1	1.2
	Farmers' group	1	1.2
	No preference	1	1.2

* Community disease reporters are community animal health volunteers who are not officially remunerated for their services but may be paid tokens by the community for their services.

** Baraza is the informal village-level dissemination fora.

**Attitudes that may increase risk perception to camel respiratory diseases**: (1) Association of climatic conditions (cold, dry, and rainy) conditions to respiratory diseases, (2) Association of age to camel respiratory conditions, and (3) Perception that camels are highly valued animals.

**Attitudes that may decrease risk perception to camel respiratory diseases**: (1) Perception that camels are hardy animals, (2) Perception that government does not care for camels, (3) Low preference given to animal health professionals as a preferred source of information, (4) Doing nothing when encountered with challenges, and (5) Self-treatment of the sick camel.

### Practices

When camels fell sick, the most preferred practice was treatment, first by the owners (71.8%). Only 21.2% of the respondents consulted an animal health service provider. Others prayed for the camels (1.2%) or did a variety of other things (5.8%). Using serial positioning to analyze this practice, the treatment by owners, isolation of sick camels, let them recover on their own, seek help from herbalists, and slaughter of sick animal were practiced in this descending order (Table 6). When faced with the challenge of feed scarcity most camel keepers migrate in search of pastures (88.4%), 7.0% buy feeds, 2.3% rent pasture fields, and 2.3% do nothing. To address water scarcity, camel farmers migrate to areas with watering points or do nothing. To overcome marketing challenges the majority (70%) sell camels at low prices, others seek government support, look for alternative markets or do nothing about it. To address the challenge of theft, camel farmers report to government authorities (41.2%), 35.3% attempt tracking and retrieval by self, and 8.8% fight back while others migrate, brand their animals, keep guard, or do nothing. Watering points, grazing, marketing, congregating of camels for security purposes, migration, and during clashes were listed as occasions that camels from different herds meet (Table 6).

**Table 6:** Practices associated with camel management in Kenya.

**Variable**	**Classification**	**Frequency**	**Percentage**
What is the most-preferred method of treating a sick camel? (*n* = 85)	Owner treat first	61	71.8
	Consult animal health service provider	18	21.2
	Pray for the camel	1	1.2
	Do a combination of practices	5	5.8
What do you do during feed scarcity? (*n* = 85)	Migrate in search of pasture	75	88.4
	Buy feeds	6	7.0
	Rent pasture field	2	2.3
	Do nothing	2	2.3
How do you overcome marketing challenge? (*n* = 83)	Sell at lower price	58	70
	Seek government support, look for alternative markets or do nothing	25	30
To address challenges of theft, what do you do? (*n* = 85)	Report to government authorities	35	41.2
	Attempt self-tracking and retrieval	30	35.3
	Fight back the invaders	7	8.8
	Others: Migrate to safer areas, brand animals, keep guard or do nothing.	13	14.7
		Serial position	
Ranking and positioning of treatment practice	Treatment by owners	1st	
	Isolation of sick camels	2nd	
	Allow the camel to recover on its own	3rd	
	Seek help from herbalists	4th	
	Slaughter of sick animal	5th	
List places where camels from different herds meet and interact (*n* = 85)	Watering points (90.6%), grazing (57.6%), marketing (36.5%), congregating of camels for security purposes (3.5%), migration (3.5%), and during clashes (2.4%)		

## Discussion

The study was carried out on the camel value chain in Kenya with respect to the anthropological context (human activities) of camel owners and herders and how these influence camel respiratory diseases and conditions spread, prevention, and control. The camel industry is male-dominated, possibly due to the cultural settings in the two counties, patriarchy in raising large animals or other unknown considerations (25, 26). Such male domination has also been seen elsewhere in Africa (27, 28). Although women play significant roles in milking, milk handling and processing, and many other routine management practices, and may contribute a large chunk of household incomes, their roles may have been downplayed by the observed male domination of the industry (25, 28). In Kenya, other studies have been carried out among camel-keeping communities including KAP for Rift Valley fever (29), brucellosis among nomadic pastoralists and non-pastoralists (30), a review of zoonotic pathogens of dromedary camels and humans (31), and for hygiene associated with camel milk among handlers (32, 33), as well as in other neighboring countries (34).

This study revealed that the major reason for keeping camels is for purposes of milk and meat production, and for income generation. This confirms previous findings that camels contribute significantly to food and nutritional security in the ASALs of Kenya (35). Almost 86% of the respondents did not complete primary education. This low literacy level within the study population is worrisome because health-related messaging by public and animal health professionals is largely literal and may not achieve its aims among these populations. It is advocated that risk communication and community engagement interventions should make use of simple pictorial representation among camel pastoralists (36). The communities have rich indigenous knowledge of camel health, based on experience garnered over time, and socialization. Interpersonal channels of communication were also identified as the most preferred source of information. It is unnecessary to discard such information. Rather, this should be utilized to improve behavioral change intervention among camel pastoral communities (37). More work needs to be done to understand the most effective forms of communication, whether pictorials will work best, or whether radio and personal messaging using community animal health workers will achieve better results.

Whereas the camel owners and herders perceived that camels are hardy animals and are hardly susceptible to diseases, the population-level risk for respiratory infections and conditions among the study camel herd was 91.7%. This perception among a significant proportion of the community (58.8%) can negatively affect the health-seeking behavior of camels by their keeper. It also has the potential to delay timely medical intervention for sick camels. Theory and empirical evidence have demonstrated that perceptions of risk play a key role in motivating people to adopt healthy behaviors (38–40). People who are positively optimistic are likely to have a lower risk perception index and consider themselves at a lower risk of a disease outcome (41). They are thus unlikely to seek medical attention. This could also apply to camel farmers/keepers. The communities perceive camels as a neglected domestic animal by the government and that government veterinary services are out of reach for most of them. This finding further reduces effective response by the camel owners. This also explains the administration of antimicrobials by the camel keepers instead of seeking for professional assistance on animal health from veterinarians.

Although not statistically significant, lack of education, large-sized herd, and being a herder posed risk of infection with respiratory conditions to camels. These factors as well as poverty have been identified as significant risks in zoonotic infections to humans and animals (42, 43). Associated with these findings, a variety of diseases were identified as the most important constraint to camel value chain development in parts of Kenya, and the lead cause of those diseases was pests and microorganisms. This finding is quite relevant in view of the challenges of accessing animal health services by these herders and camel owners. It has previously been reported that the diagnosis and treatment of sick animals by the owners and herders is practiced widely among the pastoralist communities, similar to the findings in our study (44). Seeking the assistance of herbalists, community disease reporters and occasionally animal health service providers was also common, and a few of the pastoralists reported slaughtering sick camels as a last resort. Furthermore, a significant number of camel keepers “*do nothing*” in response to animal health challenges. A “*do nothing*” response probably shows apathy, ignorance, or genuine discouragement due to a lack of support as far as camel health is concerned. It is plausible that these practices mentioned in the study were rampant because accessing professional veterinary services were difficult for these camel owners and herders.

The perceived hardiness of camels, which may delay reporting as explained above, may be associated with inconsistent clinical signs (e.g., coughing was observed in 85.7%, nasal discharge in 59.7%, fever in 23.4%, loss of appetite in 20.8%, and enlarged lymph nodes in 19.5% of the cases in camels, with variation across individuals and villages). Although we did not observe any consistent pattern (pathognomonic sign) with regard to these observed signs, perhaps, a clear categorization with regard to the signs and symptoms may have prevailed if these respiratory conditions were disaggregated by age, gender, and physiological conditions. It is also noted that most respiratory conditions present as respiratory complexes which may involve a number of respiratory pathogens (45, 46). As observed by the respondents, a high prevalence of respiratory diseases was associated with rainy and cold seasons and younger camels. Gardner et al. (47) have earlier confirmed the effect of these seasons on camel respiratory diseases. It should be noted that most of these camels are not housed in a proper shelter and are therefore exposed to inclement weather, especially during the rains and cold seasons, and the young animals are more affected by these conditions because they are likely to be more hypothermic and susceptible to physiologic stress (48, 49). This disposition that the extremes of weather are inimical to animal respiratory health is a positive finding because it can increase the risk perception of camel respiratory diseases and provide the basis for mitigation (50). Such positive views can be reinforced and linked with improved risk perceptions and knowledge of the importance of early diagnosis and treatment by veterinary practitioners.

Elders and herbalists were the most preferred source of camel health information based on respondents' feedback. It becomes relevant for animal health services providers (veterinarians/animal health assistants and agro-veterinary shop owners) to partner with these primary sources of information to disseminate information on risks, animal health, and good farming practices using local languages and community radio stations. Such partnerships may trigger behavior change intervention in animal health services in the ASALs.

Some of the identified practices associated with camel management in the ASALs of Kenya are important considerations for the improvement of the camel value chain, public and animal health management, and human conflict resolution. Firstly, owners treat sick animals first before consulting animal health service providers. This has the implication for the abuse of antimicrobials with potential passage to the human food chain. In addition, during feed scarcity or security challenges, most herders prefer to migrate in search of pasture. This particular practice has significant potential for herders—crop farmer conflicts, an issue that has been identified regularly in sub-Saharan Africa (51–53). In addition, some farmers do nothing or sell such sick camel at lower prices. It is likely that such camels may be slaughtered and served to humans and may introduce zoonotic or food-borne diseases to humans. Thirdly, owners–herders' attempts at self-tracking and retrieval of rustled or stolen camels and the practice of fighting back invaders are long associated with animal rustling, with unnecessary wasting of human lives. It becomes necessary that service delivery for crime reportage should be brought closer to these communities to reduce potential human conflicts associated with securing the stock. Finally, a number of high-risk areas have been identified including the watering points, the grazing areas, and the markets. The provision of necessary infrastructure services such as water, designated grazing areas, and bio-secure markets will positively impact on reducing the burden of camel diseases in the ASALs.

## Conclusion

We have identified relevant knowledge, attitudes, perceptions, and practices of camel owners and herders on camel health. These identified knowledge, attitudes, and practices should serve as entry points in creating attitudinal and behavioral change in camel health. Similarly, the animal health authorities should strive to be more responsive to the needs of camel pastoral communities in Kenya to reduce the potential burden of zoonoses and food-borne illnesses associated with camel. Development of specific communication strategy that targets the camel pastoralist communities is recommended for implementation.

## Data availability statement

The original contributions presented in the study are included in the article/Supplementary material, further inquiries can be directed to the corresponding author/s.

## Ethics statement

Ethical review and approval was not required for the study of human participants in accordance with the local legislation and institutional requirements. Written informed consent from the participants was not required to participate in this study in accordance with the national legislation and the institutional requirements.

## Author contributions

Concept: ET, MA, TN, SV, and EG. Fieldwork: JO, SM, BM, PK, BA, ET, and TN. Analysis: FF, SM, TN, and ET. Project administration: ON, MA, JM, HH, FF, and SV. Contributed resources: ON, MA, SV, JM, LM, HH, TN, and FF. Writing of initial draft: JO, SM, MA, ET, TN, and FF. All authors writing and review of the final version.

## Funding

This survey was supported by the Food and Agriculture Organization of the United Nations (FAO) in collaboration with the Director of Veterinary Services and the Isiolo and Garissa County Governments. The field work and article publication fee were covered through funding from the United States Agency for International Development (USAID) through the project ‘MERS-CoV applied research activities in the Middle East and Northeast Africa’, Project Code: OSRO/GLO/505/USA.

## Conflict of interest

The authors declare that the research was conducted in the absence of any commercial or financial relationships that could be construed as a potential conflict of interest. The funder and FAO did not influence the outcome of the research or its publication, and cannot be held liable for the outcomes.

## Publisher's note

All claims expressed in this article are solely those of the authors and do not necessarily represent those of their affiliated organizations, or those of the publisher, the editors and the reviewers. Any product that may be evaluated in this article, or claim that may be made by its manufacturer, is not guaranteed or endorsed by the publisher.
