# Supplementary material for: Knowledge, attitudes, and practices on camel respiratory diseases and conditions in Garissa and Isiolo, Kenya
Source: Front Vet Sci. 2022 Nov 29;9:1022146. doi: 10.3389/fvets.2022.1022146 (PMC9745045; doi:10.3389/fvets.2022.1022146)
Supplement: Supplementary file 1 [file Table_1.DOCX]

**MERS-COV and MERS-COV-like conditions in Camels in Isiolo and Garissa**

This questionnaire is designed for the Camel farmers (owners, herders)

**(Mark in the appropriate box)**

**Section A: Demographics (of the respondent)**

| 1. **County** | 1. **Sub County** | 1. **Ward** | 1. **Sub Location** | 1. **Village** |
| --- | --- | --- | --- | --- |
|  |  |  |  |  |
| 1. **GPS Coordinates** | 1. **Name of the interviewer** | 1. **Date of the Interview: DD/MM/YY** |  | |
|  |  | **____/_____/____** |  | |
| 1. **Sex of respondent** | 1. **Age of respondent** | 1. **Respondents education level** | | 1. **Religion of respondent** |
| 1=Male  2=Female | 1 = 18- 25  2 = 26- 35  3 = 36-45  4 = 46-55  5 = 56-65  6 = 66 > | 1 = None/ Never been to school  2 = Primary School incomplete  3 = Primary School Complete,  4 = Secondary school incomplete  5 = Secondary school Complete  6 =Tertiary incomplete  7 = Tertiary complete | | 1 = Christian  2 = Muslim  3 = Hindu  4 = Other…specify ……….. |
| 1. **Herd size** | 1. **Respondent type** |  | | |
| 1 = Small (1-5)  2 = Medium (6-30)  3 = Large > 30 | 1 = Owner  2 = Herder |  | | |

**Section B: Knowledge**

(This section assesses the knowledge levels on MERS-COV and MERS-COV-like conditions in Camels)

1. **What benefits do you get from camels?**

|  |  |  |  |
| --- | --- | --- | --- |
|  |  |  |  |
|  |  |  |  |

1. **What are the main constraints to camel farming? (Question 16 and 17 tests serial position effect so write against the list below in the order in which they are uttered by the farmer)**

|  | No. |  | No. |  | No. |  | No. |
| --- | --- | --- | --- | --- | --- | --- | --- |
| Diseases/Health |  | Feeds |  | Marketing |  | Theft |  |
| Others …………..  (specify) |  | Others …………..  (specify) |  | Others …………..(specify) |  | Others …………..  (specify) |  |

1. **What are the main diseases (syndromes) of camels? (may be given using names of specific systems affected and preferably in local names)**

|  | **Local Name** | **Scientific name** |
| --- | --- | --- |
| **1** |  |  |
| **2** |  |  |
| **3** |  |  |
| **4** |  |  |
| **5** |  |  |

1. **What causes these diseases?**

| **Pests (name them)**  **………………………………** | **Micro-organisms**  **……………………………..** | **Injuries (where and how?)…………………………** |
| --- | --- | --- |
| **Others specify ……………….** | | |

1. **Has your herd ever suffered from a respiratory disease?**

| **Yes** | **No** |
| --- | --- |

1. **If yes in question 19 above; how did the disease present (what were the clinical signs)**

|  |  |
| --- | --- |
|  |  |
|  |  |
|  |  |

1. **Did the animal die or recover from these clinical signs?**

| Recovered | Died |
| --- | --- |

1. **If the animal died, after how many days did it die after the onset of signs?**

|  |
| --- |

**Section C: Attitudes**

(This section tries to externalize covert feelings of the farmers in regards to MERS-COV and MERS-COV-like conditions in Camels)

1. **Camels are hardy animals and rarely fall sick**

| True | False |
| --- | --- |

1. **In your opinion respiratory diseases are common in which category of camels? (the answers can be young/old, lactating or all ages)**

|  |  |  |  |
| --- | --- | --- | --- |

1. **In your opinion respiratory diseases are common during? (the answers can be seasonal – rainy, dry or cold, or cultural occasions)**

|  |  |  |  |  |
| --- | --- | --- | --- | --- |

1. **Is there anybody with traditional knowledge (herbalist) on camel diseases within your locality?**

| YES | NO |
| --- | --- |

1. **If yes, have you ever gotten information from him/her?**

| YES | NO |
| --- | --- |

1. **Do you have a vet/animal health assistant within your locality? (you can have more than one answer)**

| YES | Vet……... | AHA……………… | Disease Reporter ………… | NO …… |
| --- | --- | --- | --- | --- |

1. **Who is most accessible?**

| Vet ………. | AHA……………. | Disease Reporter ………. | Herbalist ………. |
| --- | --- | --- | --- |

1. **What is your preferred source of information on camel health issues?**

………………………………………………………………………………………………………………

1. **How much support is government giving camel farmers to maintain good health, productivity and trade?**

| Sufficient support | Little support | No support | I do not know |
| --- | --- | --- | --- |

**Section D: Practices**

1. **How do you address constraints listed in question 16?**

|  | **Constraint** | **Solution** |
| --- | --- | --- |
| 1 | Diseases/Health |  |
| 2 | Feeds/nutrition |  |
| 3 | Marketing |  |
| 4 | Theft |  |
| 5 | Others ……………………………. |  |
| 6 | Others ……………………………. |  |

1. **When do you normally bring camels from different herds together? (answers can be markets, watering, games etc).**

|  |  |  |  |
| --- | --- | --- | --- |
|  |  |  |  |

1. **When your camel comes down with respiratory clinical signs, what do you do? (this question tests serial positioning effect write the numbers according to how they are uttered by the farmer.**

|  | **No.** |  | **No.** |
| --- | --- | --- | --- |
| Isolate the sick animal from the rest |  | Treat it my self |  |
| Slaughter the sick animal |  | Look for a herbalist to treat it…… |  |
| Sell it quickly to traders |  | Sell it quickly to other farmers |  |
| Look for a Vet. To treat it |  | Let it recover by itself |  |
| Wait and see if it recovers on its own |  | Others specify ……………………… |  |
| Others specify ………………... |  | Others specify …………………….. |  |

1. **Have you received information on camel health?**

| YES | NO |
| --- | --- |

1. **If yes from which sources did you receive this information from?**

|  | **No.** |  | **No.** |
| --- | --- | --- | --- |
| Interpersonal channels (specify) Barazas, fellow farmer, field days |  | Mass media (specify)  Radio, TV, Newspaper |  |
| IECs (brochures, posters, banners, booklets) |  | Veterinary doctor/AHA |  |
| Chief’s Baraza |  | Newspaper |  |
| Radio |  | TV |  |
| Internet (google) |  | Others (specify)…………………. |  |
| NGOs (Name………………………….............…..) |  | Others (specify)…………………. |  |

1. **In your opinion how has COVID19 affected camel farming**

**………………………………………………………………………………………………………………………………………………………………………………………………………………………………………………………………………………………………………………………**

1. **Is there anything important about camel health that we have not asked you?**

**………………………………………………………………………………………………………………………………………………………………………………………………………………………………**

**……………………………………………………………………………………………………………….**

**Thank you for sharing with us this information, we promise to give you feedback on this so that it can help you improve the health of your camels**

**Tel. of respondent …………………………………………………………………………………………**
