# Supplementary material for: Knowledge, attitudes, and practices on camel respiratory diseases and conditions in Garissa and Isiolo, Kenya
Source: Front Vet Sci. 2022 Nov 29;9:1022146. doi: 10.3389/fvets.2022.1022146 (PMC9745045; doi:10.3389/fvets.2022.1022146)
Supplement: Supplementary file 2 [file Table_2.DOCX]

**KEY INFORMANTS GUIDE: RESPIRATORY DISEASES IN CAMELS**

**A) Vets/Animal Health Personnel**

**County: …………………………………………………………………….…………..………**

**Sub-County: ………………………………………………………………..…………………..**

**Ward: …………………………………………………………………………………………..**

**Village (where applicable) …….…………………………………………..…………………..**

**GPS Coordinates (where applicable):…………………………………………………………**

1. **What is the state of veterinary health service in the county?**

● Personnel (vets, AHAs, paravets)

● Resources, communications, transport, cold chain, etc.

● Disease surveillance system (Disease reporting system, Active surveillance, Passive Surveillance, outbreak investigation)

1. **What are the main camel diseases in the County?**

|  |  |
| --- | --- |
|  |  |
|  |  |
|  |  |
|  |  |

1. **What are your general comments on respiratory diseases (syndromes) in camels in the county? (Seasonality of disease occurrence or outbreaks, Mortality/morbidity, Data)**
2. **What are the perceptions of the farmers on respiratory diseases in camels?**

**(do they report, do they treat, what’s the level of awareness etc)**

1. **What in your opinion are some of the farmer behaviours/practices that can contribute to spread of camel respiratory diseases in this county?**
2. **If you were to carry out an awareness campaign on camel respiratory diseases what will be the main channels of communication?**

**7. In your opinion how has COVID19 affected camel farming/farmers?**
