# Supplementary material for: Knowledge, attitudes, and practices on camel respiratory diseases and conditions in Garissa and Isiolo, Kenya
Source: Front Vet Sci. 2022 Nov 29;9:1022146. doi: 10.3389/fvets.2022.1022146 (PMC9745045; doi:10.3389/fvets.2022.1022146)
Supplement: Supplementary file 3 [file Table_3.DOCX]

**KEY INFORMANTS GUIDE: RESPIRATORY DISEASES IN CAMELS**

**Mark ‘X’ against your selection to indicate if you are answering as:**

| **Members of the Camel Association** |  |
| --- | --- |
| **Community Opinion Leader** |  |
| **A trader** |  |

**Move to the appropriate section (b, c or d) based on your answer above.**

**b) CAMEL ASSOCIATIONS**

**I) Basic Information**

**Name of the association …………………………………………………….**

**County: …………………………………………………………………..………**

**Sub-County: ……………………………………………………………………..**

**Ward: ……………………………………………………………………………..**

**Village (where applicable)…….…………………………………………………..**

**Membership (numbers and geographical coverage) ………………………………………………………………………………………**

**Main goal (s) of the association**

**……………………………………………………………………………………………………………………………………………………………………………………………………………………………………………………………………………………………………….**

1. **What are the main camel diseases?**

|  |  |
| --- | --- |
|  |  |
|  |  |
|  |  |
|  |  |

1. **What are your general comments on respiratory diseases (syndromes) in camels (Seasonality of disease occurrence or outbreaks, Mortality/morbidity)**
2. **How do farmers deal with such diseases?**
3. **What are the main concerns of camel farmers during your interactions?**
4. **As an association, how do you help camel farmers improve the health of their camels?**

**4. As an association have you ever shared information on camel health with camel farmers/members?**

**5. What are the main constraints to camel farmers according to you?**

**6. What can government do to improve the welfare of camel farmers?**

**8. What else can you say about camel farming**

**9. In your opinion how has COVID19 affected camel farming/farmers?**

**KEY INFORMANTS GUIDE: RESPIRATORY DISEASES IN CAMELS**

**c) Opinion leaders**

**I) Basic Information**

**County: …………………………………………………………………..………**

**Sub-County: ……………………………………………………………………..**

**Ward: ……………………………………………………………………………..**

**Village (where applicable)…….…………………………………………………..**

**Area of expertise/why one is an opinion leader……………………………….**

1. **What are the main camel diseases?**

|  |  |
| --- | --- |
|  |  |
|  |  |
|  |  |
|  |  |

1. **What are your general comments on respiratory diseases (syndromes) in camels (Seasonality of disease occurrence or outbreaks, Mortality/morbidity)**
2. **How do farmers deal with such diseases?**
3. **What are the main concerns of camel farmers during your interactions?**
4. **How do you help camel farmers improve the health of their camels?**

**4. As an opinion leader have you ever shared information on camel health with camel farmers or general public?**

**5. What are the main constraints to camel farmers according to you?**

**6. What can government do to improve the welfare of camel farmers?**

**8. What else can you say about camel farming**

**9. In your opinion how has COVID19 affected camel farming/farmers?**

**KEY INFORMANTS GUIDE: RESPIRATORY DISEASES IN CAMELS**

**d) Traders**

**County: …………………………………………………………………….…………..………**

**Sub-County: ………………………………………………………………..…………………..**

**Ward: …………………………………………………………………………………………..**

**Village (where applicable)…….…………………………………………..…………………..**

**GPS Coordinates (where applicable):…………………………………………………………**

**Number of years in trading ………………………………………………………………….**

**Gender………………………………………………………………………**

**Religion ………………………………………………………………………..**

**Markets coverage (which markets he/she sells or buys)……………………………………**

**Nature of business (live animals, meat or by products)…………………………………………………………………………….**

1. **Do you also keep camels?**

| **Yes** | **No** |
| --- | --- |

1. **If yes do you separate them from the ones you trade in?**

| **Yes** | **No** |
| --- | --- |

1. **If yes why do you do that?**
2. **What are the main camel diseases?**

|  |  |
| --- | --- |
|  |  |
|  |  |
|  |  |
|  |  |

1. **What are your general comments on respiratory diseases (syndromes) in camels (Seasonality of disease occurrence or outbreaks, Mortality/morbidity)**
2. **Do you consider generally the health of camels while buying or selling?**

| **Yes** | **No.** |
| --- | --- |

1. **Do you consider respiratory diseases while buying or selling camels?**

| **Yes** | **No.** |
| --- | --- |

1. **If yes why do you think this is important**

|  |  |
| --- | --- |
|  |  |
|  |  |
|  |  |
|  |  |

1. **Is there any other issue you wish to share with us on camels?**

**10. In your opinion how has COVID19 affected camel farming/farmers?**

**Thank you for the time, we shall share with you the feedback of this discussion for improvement of your trade.**
