# Supplementary material for: Knowledge, attitudes, and practices on camel respiratory diseases and conditions in Garissa and Isiolo, Kenya
Source: Front Vet Sci. 2022 Nov 29;9:1022146. doi: 10.3389/fvets.2022.1022146 (PMC9745045; doi:10.3389/fvets.2022.1022146)
Supplement: Supplementary file 4 [file Table_4.DOCX]

**KEY INFORMANTS GUIDE: RESPIRATORY DISEASES IN CAMELS**

**C) Opinion leaders**

**I) Basic Information**

**County: …………………………………………………………………..………**

**Sub-County: ……………………………………………………………………..**

**Ward: ……………………………………………………………………………..**

**Village (where applicable)…….…………………………………………………..**

**Area of expertise/why one is an opinion leader……………………………….**

1. **What are the main camel diseases?**

|  |  |
| --- | --- |
|  |  |
|  |  |
|  |  |
|  |  |

1. **What are your general comments on respiratory diseases (syndromes) in camels (Seasonality of disease occurrence or outbreaks, Mortality/morbidity)**
2. **How do farmers deal with such diseases?**
3. **What are the main concerns of camel farmers during your interactions?**
4. **How do you help camel farmers improve the health of their camels?**

**4. As an opinion leader have you ever shared information on camel health with camel farmers or general public?**

**5. What are the main constraints to camel farmers according to you?**

**6. What can government do to improve the welfare of camel farmers?**

**8. What else can you say about camel farming**

**9. In your opinion how has COVID19 affected camel farming/farmers?**
