# Supplementary material for: Knowledge, attitudes, and practices on camel respiratory diseases and conditions in Garissa and Isiolo, Kenya
Source: Front Vet Sci. 2022 Nov 29;9:1022146. doi: 10.3389/fvets.2022.1022146 (PMC9745045; doi:10.3389/fvets.2022.1022146)
Supplement: Supplementary file 5 [file Table_5.DOCX]

**KEY INFORMANTS GUIDE: RESPIRATORY DISEASES IN CAMELS**

**c) Traders**

**County: …………………………………………………………………….…………..………**

**Sub-County: ………………………………………………………………..…………………..**

**Ward: …………………………………………………………………………………………..**

**Village (where applicable)…….…………………………………………..…………………..**

**GPS Coordinates (where applicable):…………………………………………………………**

**Number of years in trading ………………………………………………………………….**

**Gender………………………………………………………………………**

**Religion ………………………………………………………………………..**

**Markets coverage (which markets he/she sells or buys)……………………………………**

**Nature of business (live animals, meat or by products)…………………………………………………………………………….**

1. **Do you also keep camels?**

| **Yes** | **No** |
| --- | --- |

1. **If yes do you separate them from the ones you trade in?**

| **Yes** | **No** |
| --- | --- |

1. **If yes why do you do that?**
2. **What are the main camel diseases?**

|  |  |
| --- | --- |
|  |  |
|  |  |
|  |  |
|  |  |

1. **What are your general comments on respiratory diseases (syndromes) in camels (Seasonality of disease occurrence or outbreaks, Mortality/morbidity)**
2. **Do you consider generally the health of camels while buying or selling?**

| **Yes** | **No.** |
| --- | --- |

1. **Do you consider respiratory diseases while buying or selling camels?**

| **Yes** | **No.** |
| --- | --- |

1. **If yes why do you think this is important**

|  |  |
| --- | --- |
|  |  |
|  |  |
|  |  |
|  |  |

1. **Is there any other issue you wish to share with us on camels?**

**10. In your opinion how has COVID19 affected camel farming/farmers?**

**Thank you for the time, we shall share with you the feedback of this discussion for improvement of your trade.**
