# Supplementary material for: Knowledge, attitudes, and practices on camel respiratory diseases and conditions in Garissa and Isiolo, Kenya
Source: Front Vet Sci. 2022 Nov 29;9:1022146. doi: 10.3389/fvets.2022.1022146 (PMC9745045; doi:10.3389/fvets.2022.1022146)
Supplement: Supplementary file 6 [file Table_6.DOCX]

**MERS-COV and MERS-COV-like conditions in Camels in Isiolo and Garissa**

**FOCUS GROUP DISCUSSIONS GUIDE**

**Participants**:

Camel farmers involved in rearing and herding of camels will be selected as participants in the focus group discussion. The participants will be categorized by age, gender, level and pattern of working (e.g. both male and female farmers).

**Facilitators/Moderators**:

- Facilitator to work with translator speaking the local language.
- Probe further based on the responses received or rephrase questions for clarity
- Keep participants focused on topic
- Allow equal participation of members
- Be very familiar with the FGD questionnaire
- Be an active listener

**The note taker**

- Work closely with the translator to record the group discussion effectively.
- Record the behavior of the participants (remarkable attitudes, spontaneous reactions, interactions among the participants
- Maintain confidentiality while recording the discussion
- Be familiar with the list of questions and the topic of investigation

**Introduction:**

- Explain the purpose of having the focus group discussion; to assess their knowledge, attitude and practices on Camel respiratory diseases
- Specify the duration of time the discussion is expected to take e.g. 30 min
- Seek permission to record the discussion to facilitate its recollection (if yes, switch on the recorder)

**Anonymity:**

- Give assurance that the discussion will be anonymous: recording will the safely locked and disposed once transcribed, transcriptions will contain no information that would allow individual subjects to be linked to specific statements
- Clearly state that there is free will to answer and decline to engage in discussions that they do not wish to answer or participate

**Ground rules**

- Only one person speaks at a time. There may be a temptation to jump in when someone is talking but please wait until they have finished
- There are no right or wrong answers
- You do not have to speak in any particular order
- You are encouraged to answer and comment as accurately and truthfully as possible
- When you do have something to say, please do so
- There are many of you in the group and it is important that I obtain the views of each of you
- You do not have to agree with the views of other people in the group
- Does anyone have any questions? (Answers). Begin

**Ice breaker**

First, everyone should introduce themselves. Can you tell us your name?

**Introductory question**

Give them a couple of minutes to think about what they know about respiratory diseases in Camels. The diseases can be listed in their mother tongue and translated. Ask if anyone would like to share with the group about what they know.

**Guiding questions**

1. What benefits do you get from camels?
2. Camels are certainly among the main domestic animals you keep, what makes camels among the best animals to keep here (prod for their attitudes on camel as a hardy animal that doesn’t require a lot of veterinary attention – try to see whether this is externalized in the discussion)
3. What are the main constraints to camel farming and how do you handle them?

|  | **Constraint** | **Solution** |
| --- | --- | --- |
| 1 | Diseases/Health |  |
| 2 | Feeds/nutrition |  |
| 3 | Marketing |  |
| 4 | Theft |  |
| 5 | Others ……………………………. |  |
| 6 | Others ……………………………. |  |
|  |  |  |
|  |  |  |
|  |  |  |

1. What are the main diseases of camels? (may be given using names of specific systems affected and preferably in local names)
2. What causes these diseases?
3. Has your herd ever suffered from a respiratory disease?
4. If yes in the question above; how did the disease present (what were the clinical signs)
5. Do you have a local name(s) for these disease(s)? How do you call it or them (there might be collective name for a number of diseases – try to externalize this.
6. Respiratory diseases are common in? – Age (old, young, all), season (dry, cold, windy, rainy)
7. When do you normally bring camels from different herds together?
8. When animals become sick what do you do?

|  | **No.** |  | **No.** |
| --- | --- | --- | --- |
| Isolate the sick animal from the rest |  | Treat it my self |  |
| Slaughter the sick animal |  | Look for a herbalist to treat it…… |  |
| Sell it quickly to traders |  | Sell it quickly to other farmers |  |
| Look for a Vet. To treat it |  | Let it recover by itself |  |
| Wait and see if it recovers on its own |  | Others specify ……………………… |  |
| Others specify ………………... |  | Others specify …………………….. |  |

1. How do you get information on animal health?

|  | **No.** |  | **No.** |
| --- | --- | --- | --- |
| Interpersonal channels (specify) Barazas, fellow farmer, field days |  | Mass media (specify)  Radio, TV, Newspaper |  |
| IECs (brochures, posters, banners, booklets) |  | Vets/AHAs/CBHWS |  |
| NGOs (Name…………………………..) |  | Others (specify) |  |

**13) In your opinion how has covid19 affected camel farming**

**Concluding question**

- Of all the things we’ve discussed today, what would you say are the most important issues?
- Do you have any other question?

**Conclusion**

- Thank them for participating. This has been a very successful discussion
- Your opinions will be a valuable asset to the study
- We hope you have found the discussion interesting
- I would like to remind you that any comments featuring in this report will be anonymous
